# The origin of glutamatergic synaptic inputs controls synaptic plasticity and its modulation by alcohol in mice nucleus accumbens

**DOI:** 10.3389/fnsyn.2015.00012

**Published:** 2015-07-20

**Authors:** Xincai Ji, Sucharita Saha, Gilles E. Martin

**Affiliations:** Department of Psychiatry, Brudnick Neuropsychiatric Research Institute, University of Massachusetts Medical SchoolWorcester, MA, USA

**Keywords:** drug addiction, electrophysiology, synaptic plasticity, optogenetics, accumbens, alcohol

## Abstract

It is widely accepted that long-lasting changes of synaptic strength in the nucleus accumbens (NAc), a brain region involved in drug reward, mediate acute and chronic effects of alcohol. However, our understanding of the mechanisms underlying the effects of alcohol on synaptic plasticity is limited by the fact that the NAc receives glutamatergic inputs from distinct brain regions (e.g., the prefrontal cortex (PFCx), the amygdala and the hippocampus), each region providing different information (e.g., spatial, emotional and cognitive). Combining whole-cell patch-clamp recordings and the optogenetic technique, we examined synaptic plasticity, and its regulation by alcohol, at cortical, hippocampal and amygdala inputs in fresh slices of mouse tissue. We showed that the origin of synaptic inputs determines the basic properties of glutamatergic synaptic transmission, the expression of spike-timing dependent long-term depression (tLTD) and long-term potentiation (LTP) and long-term potentiation (tLTP) and their regulation by alcohol. While we observed both tLTP and tLTD at amygadala and hippocampal synapses, we showed that cortical inputs only undergo tLTD. Functionally, we provide evidence that acute Ethyl Alcohol (EtOH) has little effects on higher order information coming from the PFCx, while severely impacting the ability of emotional and contextual information to induce long-lasting changes of synaptic strength.

## Introduction

The nucleus accumbens (NAc), a forebrain region that is part of the mesocorticolimbic circuitry (Humphries and Prescott, 2010), has long been identified as a key structure mediating the rewarding aspect of addiction (Feltenstein and See, 2008). On the basis of cytoarchitectonic and immunohistochemical criteria, this region is divided between the core and shell, with the former resembling the dorsal striatum, and the latter typically being associated with the amygdala (Groenewegen et al., 1999). There is strong evidence that long-lasting adaptations of the strength of synaptic connections are responsible for the addictive properties of alcohol (Luscher and Malenka, 2011; Zorumski et al., 2014). Unfortunately, the cellular and molecular mechanisms underlying synaptic plasticity in this brain region remain ill-defined in part due to difficulties in evoking reliable long-term potentiation (LTP; Pennartz et al., 1993; Robbe et al., 2002), the form of synaptic plasticity associated with learning and memory, and more generally with the ability of the brain to adapt to changing conditions (Letzkus et al., 2007). Further hampering progress is the fact that both core and shell accumbens receive glutamatergic afferents from different brain regions [e.g., Prefrontal cortex (PFCx), amygdala, thalamus and hippocampus; Humphries and Prescott, 2010], each pathway carrying different types of information to NAc medium spiny neurons (MSNs). While signals sent by the PFCx carry information associated with higher form of executive/cognitive processing, those sent by the amygdala and hippocampus inform the accumbens about the emotional state and contextual information, respectively. Thus far, the traditional approach consisting of evoking synaptic plasticity by electrical stimulation does not distinguish between these three pathways, making it impossible to address basic questions relative to how MSNs process cortical, amygdala and hippocampal information. Of particular importance is how similar the basic properties of these synapses and their ability to undergo plasticity are. Also unknown is whether the origin of inputs is a determining factor shaping the action of alcohol on synaptic plasticity. We combined *in vitro* whole-cell patch clamp recordings in brain slices with using the optogenetic technique to evoke excitatory postsynaptic potentials (EPSPs) from specific pathways and to examine their properties as well as the characteristics of synaptic plasticity in core NAc MSNs. Also, to circumvent limitations usually associated with high frequency stimulation, we used spike-timing dependent plasticity (STDP), a stimulation paradigm based on the pairing of action potential (AP) and EPSPs at low frequency (Feldman, 2012), mimicking NAc MSNs *in vivo* low firing rate (~1–5 Hz; Chang et al., 1994; Ishikawa et al., 2008), to reliably evoke tLTP and timing dependent long-term depression (tLTD) (Ji and Martin, 2012). Our study reveals that various aspects of glutamatergic synaptic transmission (i.e., AMPA/NMDA ratio, NMDA sensitivity to magnesium, probability of release) present distinct characteristics based on its origin. We also show that the origin of afferents determines the nature of synaptic plasticity, with tLTP being absent at cortical synapses but not at amygdala and hippocampal inputs. Finally, we report that a low alcohol concentration (i.e., 20 mM) fully inhibits tLTP at amygdala and hippocampal inputs. In contrast, tLTD at PFCx, amygdala and hippocampal synapses remains mostly unaffected by the drug.

## Materials and Methods

All experiments, with the exception of one where male B6.Cg-Tg (Drd1a-tdTomato) were used (inset, Figure 3A), were performed on male wild type (wt) and BK channel β4 knockout C57Bl/6J mice. All mice were handled according to the American Association for the Accreditation of Laboratory Animal Care guideline. The protocol was approved by the Institutional Animal Care and Use Committee of University of Massachusetts Medical School. Mice were maintained at constant temperature and humidity with a 12-h light–dark cycle. Water and food were provided ad libitum.

### Animal Surgeries and Slice Preparation

We injected 21- to 24-day-old (10–15 g) C57Bl/6J mice with adeno-associated virus containing ChR2–YFP (AAV9 EF1α-hChR2-(H134R)-EYFP) bilaterally (0.8 μl in each side) using a Hamilton syringe with a 2″ long 26g needle stereotaxically placed into the prelimbic PFCx (Figure 2A; anteroposterior, +0.32 cm from bregma; mediolateral, 0.06 cm from bregma; ventral, −0.42 cm from skull surface), the amygdala basal nucleus (Figure 2D; −0.23 cm; 0.32 cm; −0.42 cm), or the ventral hippocampus (Figure 2H; −0.18 cm; 0.32 cm; −0.42 cm). Virus injections in the PFCx, amygdala and hippocampus were carried out on different mice. The injector was left in place for 5 min, raised 1 mm, and left for an additional 5 min before being removed. We returned mice to their home cages for 21 days before performing electrophysiological experiments. To prepare coronal slices from fresh brain tissue, that contains only terminals of neurons originating from the various brain region mentioned above, we rapidly removed and transferred the brain in a cold (~+0.5°C) oxygenated (95% O_2_ and 5% CO_2_) of the following composition (in mM): 95 N-methyl-D-glucamine (NMDG), 2 thiourea, 5 Na^+^-ascorbate, 3 Na^+^-pyruvate to cut slices (300 μm) with a Vibroslicer (VT1200, Leica MicroInstrutments; Germany). Slices were immediately transferred in an incubation chamber and left to recuperate in the NMDG-based solution for 22 min at 32°C before being moved into a chamber containing an artificial cerebrospinal fluid (ACSF; in mM): 126 NaCl, 2.5 KCl, NaH_2_PO_4_.H_2_O, 1 MgCl_2_, 2 CaCl_2_, 26 NaHCO_3_, 10 D-Glucose, at room temperature. Slices were left in this chamber for at least 1 h before being placed in a recording chamber and perfused with ACSF at a constant rate of 2–3 ml/min at room temperature (~21°C). We visualized neurons in infrared differential interference contrast (60×, IR-DIC) videomicroscopy using a fully motorized upright microscope (Scientifica; England).

### Electrophysiology

We performed whole-cell patch clamp recordings as described elsewhere (Ji and Martin, 2012). Briefly, following seal rupture, series resistance (Rs), typically ranging between 10 and 20 MΩ, was fully compensated in current-clamp recording mode, and periodically monitored throughout recording sessions. Recordings with Rs changes larger than 20% were rejected. We acquired voltage and current traces in whole-cell patch-clamp with an EPC10 amplifier (HEKA Elektronik, Germany). When recording AMPA- and NMDA-EPSCs in voltage-clamp mode, we used Cs^+^-methanesulfonate and added 2 mM QX-314 in the recording pipette solution. We sampled and filtered voltage and current traces acquired with PatchMaster 2.15 (HEKA Elektronik, Germany) at 5 kHz, and 2 kHz respectively. When studying action potentials (APs), voltage traces were sampled at 10 kHz. We subsequently analyzed all traces off-line using FitMaster 2.15 (HEKA Electronik, Germany). We evoked EPSPs-Cs by flashing 470 nm blue light (0.5–1 ms) through the light path of a microscope 60× objective using a high-powered LED (pE-100 CooLED, NY, USA) under the control of the acquisition software (PatchMaster, HEKA, Germany). To generate synaptic plasticity, we paired postsynaptic AP (evoked with a 5 ms/6–800 pA depolarizing pulse) and EPSP with a 20 ms interval at a rate of 1 Hz for 90 s as described previously (Ji and Martin, 2012). When examining AMPA NMDA ratio in voltage-clamp mode, we evoked AMPA-EPSCs at −70 mV in presence of 15 μM bicuculline (BIC). When EPSPs were totally blocked 3–4 min after DNQX perfusion, we held membrane potential at +40 mV to evoke NMDA-EPSCs, before changing holding potential to measure the sensitivity of NMDA receptor to magnesium. We purchased QX-314 from Ascent Scientific (Cambridge, MA, USA), BIC, spermine and DNQX from Sigma-Aldrich (Saint Louis, MO, USA).

### Analysis

tLTP/tLTD: We compared EPSPs maximum amplitude measured in a 20 ms time window 10 ms before and after the onset of the stimulus. We performed this measurement on 30 consecutive EPSPs before, and 20 min after AP-EPSP pairing to smooth out the natural variability of EPSPs amplitude. We expressed the difference of EPSP amplitude before and after induction as percent of control (100%). Statistical analysis, in Prism 5 (Graphpad, CA, USA) running on a Mac Power PC G5, was performed with Student’s one sample or unpaired *t*-tests, with *p* < 0.05 considered statistically significant. All averaged results are expressed as mean ± SEM values. AMPA NMDA ratio and paired-pulse ratio (PPR): we studied PPR by eliciting AMPA-EPSCs in presence of 15 μM BIC at −70 mV. Intervals between paired EPSC ranged from 50–200 ms with 50 ms increments. For each interval we recorded five consecutive traces every 10 s We measured the probability of release by measuring the ratio of the amplitude of the second over the first EPSC at every intervals.

## Results

### The Origin of Afferents Determines Synaptic Plasticity

**Figure 1:**
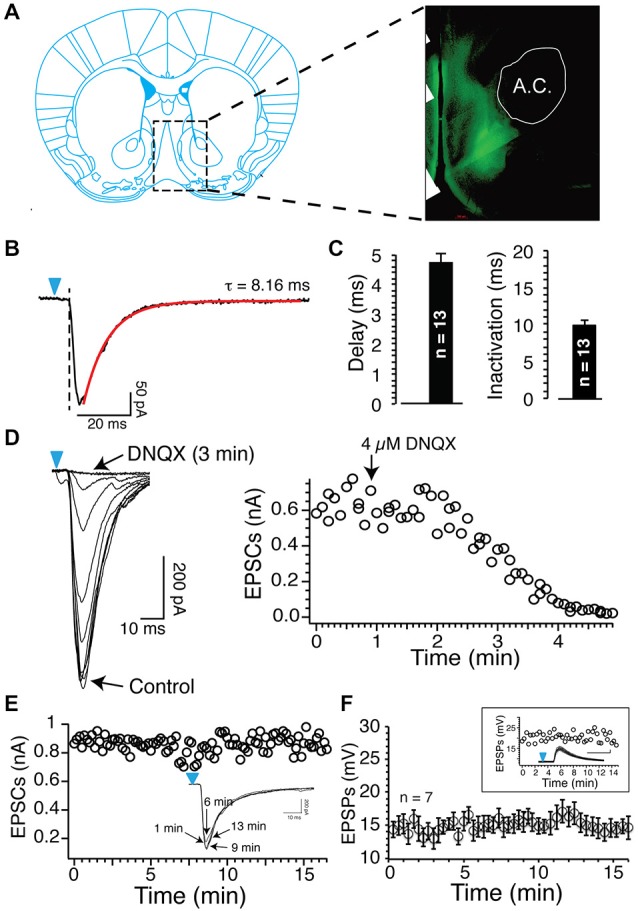
**Blue light reliably evokes excitatory postsynaptic currents (EPSCs) and EPSPs. (A)** Epifluorescence image of ChR2-expressing axons from glutamatergic cortical neurons (a.c. anterior commissure). **(B)** Inward currents evoked by short (1 ms) light stimulation in Nucleus accumbens (NAc) MSNs following injections of 0.8 μl AAV9 in the prelimbic prefrontal cortex (PFCx). Note the delay between the onset of light pulse (blue arrowheads) and the inward current. Current deactivation is fitted with a single exponential (red line). **(C)** Average delay and deactivation rates of inward currents (dark bars, synaptic EPSCs) recorded after injections of 0.8 μl AAV9. Values are represented as mean ± SEM. **(D)** DNQX (4 μM) total blocked inward currents evoked following stimulation of PFCx afferents. Graph shows the time course of DNQX inhibitory effect. **(E)** Amplitude of EPSCs evoked by stimulating cortical afferents every 20 s over a period of 15 min. From same neuron, overlapping EPSCs recorded at different times. **(F)** Average EPSPs amplitude evoked in seven MSNs over a 15 min recording session.

In a previous study, using electrical stimulation to recruit all afferents irrespective of their origin, we showed that AP-EPSP pairing evoked both tLTP and tLTD, and that these two forms of plasticity were controlled by two distinct mechanisms, NMDA receptors and APs, respectively (Ji and Martin, 2012). Here, using blue light that reliably evoked DNQX-sensitive EPSCs/EPSPs (Figures 1B–D), we examined the respective roles of PFCx, the amygdala and hippocampus inputs in shaping synaptic plasticity. First, as a control, we showed that stimulation of cortical afferents every 20 s for 15 min in absence of induction evoked stable EPSCs amplitude (individual example; Figure 1E) and EPSPs (Figure 1F), indicating that changes following the pairing of APs and EPSPs did not result from a random change of the strength of the synaptic transmission.

**Figure 2:**
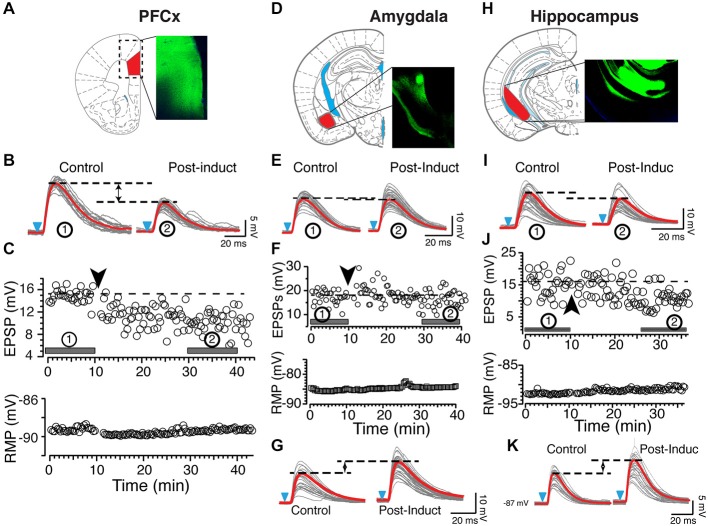
**Origin of synaptic inputs controls synaptic plasticity. (A)** Expression of ChR2 in the prelimbic area of the PFCx. Diagram and epifluorescence image of coronal section of mouse brain indicating area expressing AAV9-ChR2. **(B)** Light gray overlapping traces show 30 consecutive EPSPs evoked by stimulating afferents from the PFCx in a representative NAc MSN. EPSPs were recorded at resting membrane potential (−89 mV) before (control) and after (post-induction) AP-EPSP pairing. Horizontal dashed lines illustrate a clear depression of the synaptic strength following induction, and solid red traces indicate the average EPSPs amplitude. **(C)** In same neuron as in panel **(A)**, top and bottom graphs show EPSPs amplitude and resting membrane potential (bottom graph) monitored every 20 s before and after synaptic plasticity, respectively. Solid bars accompanied by numbers in circles in top graph indicate where EPSPs shown in panel **(A)** were recorded. **(D)** Expression of AAV9-ChR2 in the amygdala area. **(E)** Light gray overlapping traces show 30 consecutive EPSPs evoked by stimulating amygdala inputs in a different NAc MSN. EPSPs were recorded at resting membrane potential (−86 mV) before (control) and after (post-induction) AP-EPSP pairing. Broken lines illustrate the weak decrease of the synaptic strength following induction. **(F)** In same neuron as in panel **(C)**, graphs show EPSPs amplitude and the stable resting membrane potential (bottom graph) monitored every 20 s before and after synaptic plasticity. Solid bars accompanied by numbers in circles in top graph indicate when EPSPs shown in panel **(A)** were recorded. **(G)** In another MSN, stimulation of amygdala inputs induces TLTP (LTP). **(H)** Expression of AAV9-ChR2 in the ventrolateral hippocampus. Panels **(I−K)** show tLTD and tLTP at hippocampal synapses, respectively. Black and blue arrowheads indicate the time of induction and onset of light stimulation, respectively. Red lines indicate averaged EPSP amplitudes.

**Figure 3:**
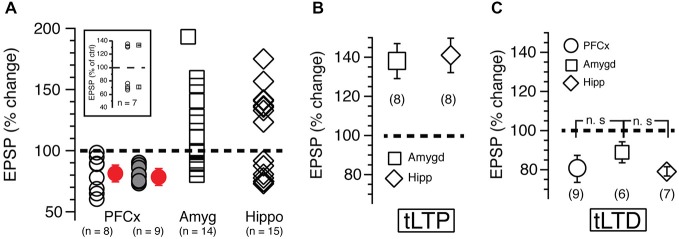
**(A)** Each open symbol, representing an individual MSN, indicates changes of EPSP amplitudes expressed as percent of control and measured 20 min after induction. Symbols above and below broken line show tLTP and tLTD, respectively. Change of synaptic strength after induction is expressed as percent of control (100%). Open circles, squares and diamonds indicate EPSPs amplitude following stimulation of cortical, amygdala and hippocampal afferents, respectively. Red symbols with SEM show averaged overall changes. In parentheses are shown number of neurons recorded in each condition. Solid gray symbols indicate the magnitude of tLTD recorded in nine MSNs after reversing the pairing order (i.e., EPSP followed by AP) during induction of plasticity. Note that this new pairing order has no impact on plasticity. Inset shows long-term potentiation (tLTP) and timing dependent long-term depression (tLTD) in MSNs expressing dopamine D1 receptors recorded in B6.Cg-Tg (Drd1a-tdTomato) mice. **(B,C)** show respectively average tLTP and tLTD expressed as percent of control following stimulation of PFCx (circle), amygdala (square) and hippocampal (diamond) inputs.

In sharp contrast to what we observed in similar experimental conditions but with EPSPs evoked with electrical stimulation (Ji and Martin, 2012), APs-EPSPs pairing evoked by selectively stimulating the PFCx pathway totally failed to elicit tLTP but led to robust tLTD. Thus, twenty minutes post induction, EPSPs amplitudes decreased markedly compared to control (Figure 2B, right panel and Figure 2C solid bar indicated with number 2). Average tLTD amplitude was 80.41 ± 3.2% of control (*n* = 8; open symbols, Figure 3A), a value in line with what we observed previously using electrical stimulation (Ji and Martin, 2012). Surprisingly, we observed no tLTP in any of the eight MSNs tested. Because tLTP is typically observed by reversing the pairing order in most brain regions (Caporale and Dan, 2008) including the dorsal striatum (Fino et al., 2005), we tested the effects of EPSP-AP pairing on another set of nine MSNs. Again, it failed to evoke tLTP. Instead, we only observed tLTD with a similar amplitude (78.1 ± 4.3%, solid gray circles; Figure 3A). Also, we were able to record tLTP and tLTD in MSNs expressing dopamine D1 receptors, suggesting that the lack of tLTP at cortical synapses is unlikely the result of a recording bias toward MSNs expressing dopamine D1Rs or D2Rs (Figure 3A, inset). To determine whether the absence of tLTP at cortical inputs was unique to those synapses, we examined plasticity at amygdala inputs under similar conditions. We found that light stimulation of amygdala afferents elicited robust tLTP (Figure 2G) in 8 out of 14 MSNs (Figure 3B), and a weak to moderate tLTD (Figures 2E,F) in the remaining six cells (Figure 3C), an inhibition that was not statistically different (89.8 ± 5.2% of control, *n* = 4) than that at cortical synapses (Figure 3C). As with amygdala inputs, we recorded both tLTD (Figures 2I,J) and tLTP (Figure 2K) when stimulating afferents originating in the hippocampal region (*n* = 15). Although a trend was clearly visible, difference of tLTD amplitude at amygdala and cortical synapses was not statistically significant (*p* = 0.098; *F*_(3,8)_ = 1.866). A similar lack of significance was observed when comparing amygdala and hippocampal tLTD amplitude (*p* = 0.076; *F*_(3,4)_ = 1.56). Taken together, these data demonstrate that plasticity at glutamatergic cortical, hippocampal and amygdala synapses is heterogenous.

### Are Differences in Plasticity Explained by Properties of Glutamatergic Synaptic Transmission?

**Figure 4:**
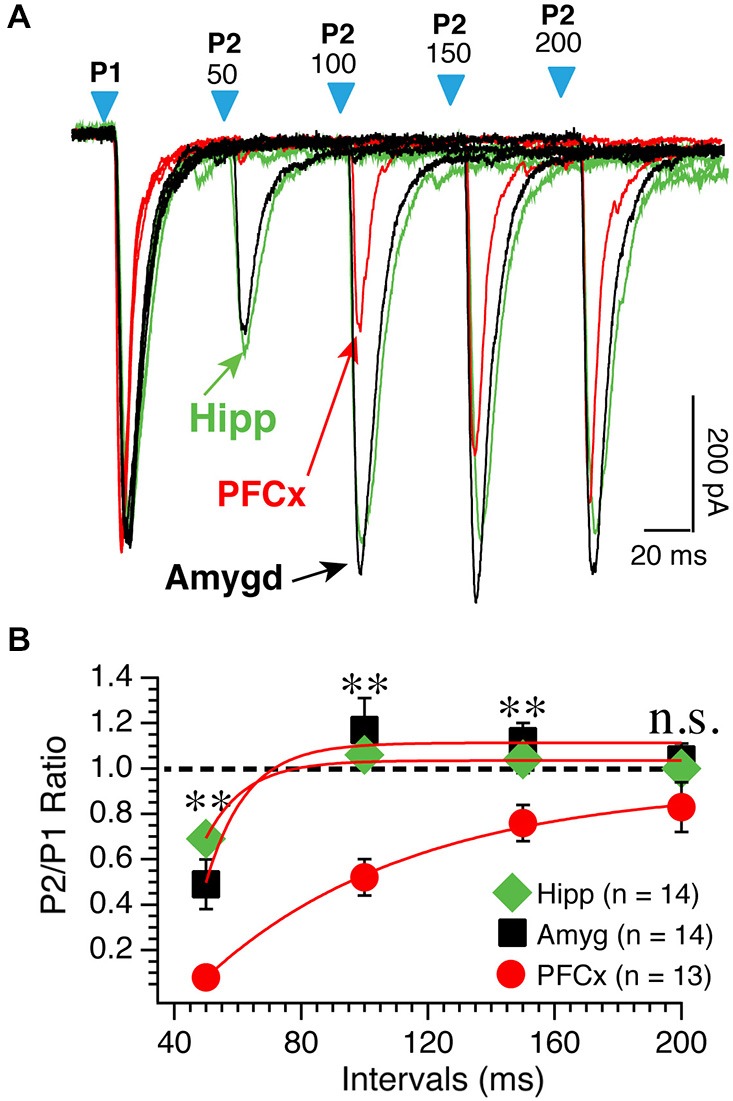
**PPR varies as a function of synaptic inputs.** **(A)** Representative overlapping individual traces of paired AMPA currents recorded at −70 mV at different intervals from 50−200 ms following stimulation of cortical (red traces), amygdala (black traces), and hippocampal (green traces) pathways. Blue arrowheads show the onset of paired light stimulations. Numbers above indicate the intervals between first and second stimulations. Hippocampal traces were scaled down to match amplitudes of similar cortical and amygdala first EPSCs. **(B)** Average P2/P1 AMPA current ratio at various intervals. *p < 0.05 and **p < 0.01.

We tested the idea that distinct basic synaptic properties accounted for the stark differences in synaptic plasticity at cortical, amygdala and hippocampal inputs. First, we examined the properties of the different pathways by assessing their respective probability of release. Thus, we measured paired AMPA currents evoked at different intervals at a holding potential of −70 mV and calculated the ratio EPSCs2/EPSCs1 (P2/P1). When stimulating cortical inputs with an interval of 50 ms, the second response was nearly absent (red traces; Figure 4A). On average, amplitude of second EPSCs was 7% of the amplitude of the first EPSCs (Figure 4B). Then, ratio increased steadily with increasing intervals (Figure 4B, red symbols). Stimulation of both amygdala and hippocampal inputs led to a very different pattern as EPSCs were readily observed at 50 ms intervals (Figure 4A, green and red traces). With longer intervals, second EPSC amplitude increased to reach that of the first ESPCs. The difference of P2/P1 ratio measured at PFCx and amygdala/hippocampus synapses was highly significant at all but the longest intervals (Figure 4B). These data contrast sharply with findings in the shell NAc (Britt et al., 2012).

**Figure 5:**
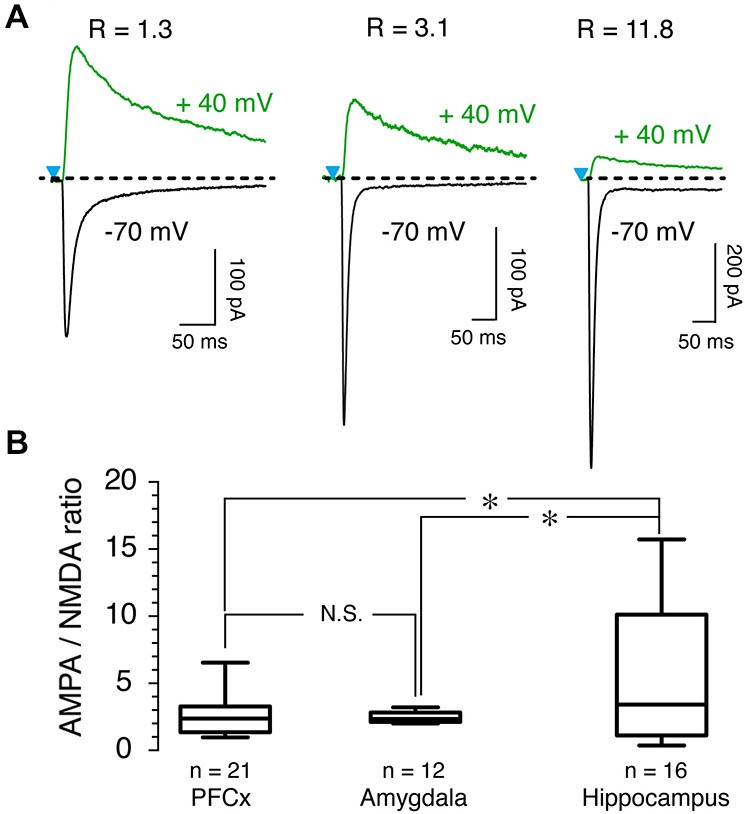
**AMPA/NMDA ratio as a function of synaptic inputs. (A)** AMPA and NMDA traces recorded at −70 mV (black traces) and +40 mV (green traces), respectively, in presence of 15 μM bicuculline (BIC) in three representative MSNs. R values above traces indicate AMPA/NMDA ratio for each neuron. **(B)** Graph shows distribution and averaged ratio for each pathway.

To further probe the characteristics of glutamate synaptic transmission of the three inputs, we measured AMPA/NMDA ratio. Although it varied widely, with values ranging from 1 up to 15 as shown by representative examples across all three brain regions (Figure 5A), we observed notable differences between inputs. While AMPA/NMDA ratio varied little from cell to cell at amygdala inputs, with an averaged value of 2.51 ± 0.1 (*n* = 12, Figure 5B), its variability was much larger at hippocampal synapses where it ranged between 0.5–15, with a mean of 5.7 ± 1.4 (*n* = 16, Figure 5B). At PFCx synapses, AMPA/NMDA ratio distribution fell between that observed at the amygdala and the hippocampus inputs, with a average of 2.51 ± 0.28 (*n* = 22). Statistical analysis between the three regions showed a significant difference between PFCx and amygdala compared to the hippocampus (*p* = 0.043, *F*_(13,21)_ = 15.27 and *p* = 0.037, *F*_(13,10)_ = 138.9 respectively).

**Figure 6:**
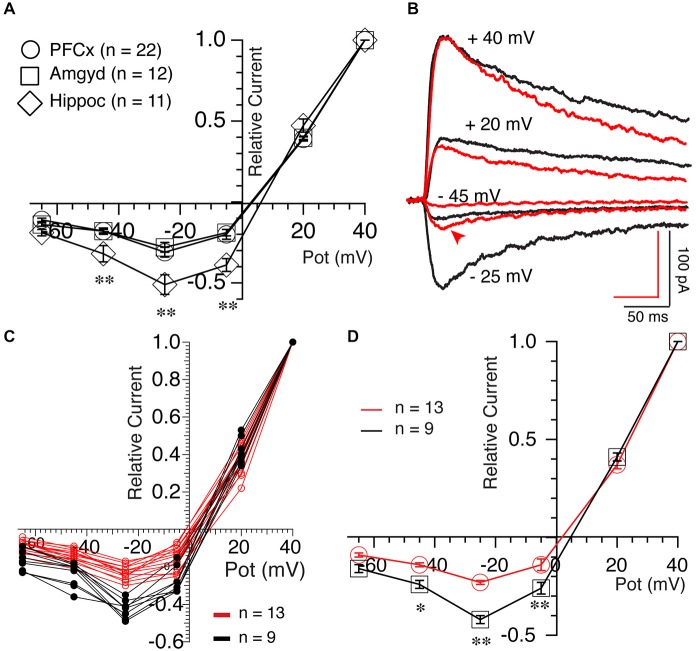
**Synaptic inputs determine NMDA receptor properties. (A)** Averaged NMDA current amplitude measured at different holding potentials between +40 and −65 mV following stimulation of PFCx (circles), amygdala (squares) and hippocampal (diamonds) afferents. Currents were expressed relative to currents recorded at +40 mV. Notable is the weaker blockade of NMDA currents by magnesium in MSNs receiving inputs from hippocampus. **(B)** NMDA currents evoked at four different potentials in presence of 4 μM DNQX and 15 μM BIC in two MSNs, illustrating their different sensitivity to magnesium at PFCx inputs. Also notable is the difference in kinetics between red and black traces at +40 mV. **(C)** Individual current-voltage relationships of NMDA currents evoked following light stimulation of cortical inputs in 22 MSNs. Red and black lines indicate NMDA currents with strong and weak sensitivity to magnesium, respectively. **(D)** Average amplitudes of NMDA current shown in graph **(C)**. *p < 0.05 and **p < 0.001.

We then examined the properties of NMDA-mediated synaptic transmission by measuring Mg^2+^-mediated blockade of NMDA currents, a property sensitive to channel subunit composition (Cull-Candy et al., 2001). In average, NMDA currents elicited both at PFCx (open circles, *n* = 22) and amygdala (open squares, *n* = 12) synapses were similarly strongly blocked by Mg^2+^ (Figure 6A). In contrast, Mg^2+^-mediated blockade at hippocampal synapses was consistently weaker (Figure 6A, open diamonds, *n* = 11). Thus, relative NMDA currents measured at −25 mV at PFCx (−0.31 ± 0.03) and amygdala (− 0.28 ± 0.03) synapses were significantly smaller (*F*_(10,25)_ = 6.14, *p* = 0.026 and *F*_(10,11)_ = 4.11, *p* = 0.004, respectively) compared to hippocampal inputs (−0.51 ± 0.06), mirroring findings in the shell NAc (Britt et al., 2012). Interestingly, upon close analysis, MSNs receiving PFCx inputs could easily be separated in two groups, one strongly blocked by Mg^2+^ and the other with less sensitive NMDA receptors as shown by representative examples in Figure 6B and in graph of Figure 6C, indicative of a heterogeneous population. At −25 mV, the relative current of the group less sensitive to Mg^2+^ was −0.45 ± 0.06 (Figure 6D, black symbols, *n* = 9) while the group the most sensitive had a relative current of −0.24 ± 0.01 (Figure 6D, red symbols, *n* = 13), a statistically significant difference (*F*_(9,12)_ = 15.89, *p* = 0.0007).

### Synaptic Pathways Control the Modulation of Synaptic Plasticity by EtOH

**Figure 7:**
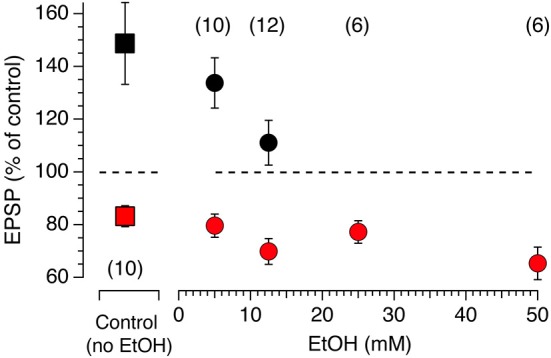
**Acute EtOH primarily targets electrically-driven tLTP in a dose-dependent fashion.** Amplitude of tLTP (solid black symbols) and tLTD (solid red symbols) in absence (solid squares, control) and presence (solid circles) of EtOH concentrations ranging from 5–50 mM. Symbols above and below the dotted line indicate tLTP and tLTD, respectively. Number of neurons recorded in each condition are indicated in parentheses. All values are represented as mean ± SEM.

Modulation of accumbens tLTP by alcohol is rarely studied and poorly understood, in part due to difficulties to reliably evoke LTP when conventional tetanic stimulation is used. Even less is known about the role of each synaptic input in mediating the effects of Ethyl Alcohol (EtOH) on synaptic plasticity. We wondered whether the heterogeneity of glutamate inputs influenced modulation of synaptic plasticity by EtOH. We first tested the effects of various concentrations of EtOH on STDP tLTP and tLTD evoked by electrical stimulation as a means to identify the optimal concentration for optogenetic experiments. In absence of EtOH, as shown previously (Ji and Martin, 2012), AP-EPSPs pairing evokes both tLTP and tLTD with an average amplitude of about 80 and 150%, respectively (Figure 6, red and black squares, respectively). In presence of a very low concentration 5 mM EtOH concentration, the inhibitory effect of the drug on tLTP, although rather small, was already visible (Figure 7, solid black circle) while it failed to affect tLTD (Figure 6, solid red circle). As EtOH concentrations increases to 12.5 mM, its inhibitory effects on tLTP become more pronounced. Finally, tLTP is totally blocked at 25 mM. In contrast, tLTD amplitude increases slightly at 12.5 and 50 mM while it barely changed at 25 mM.

**Figure 8:**
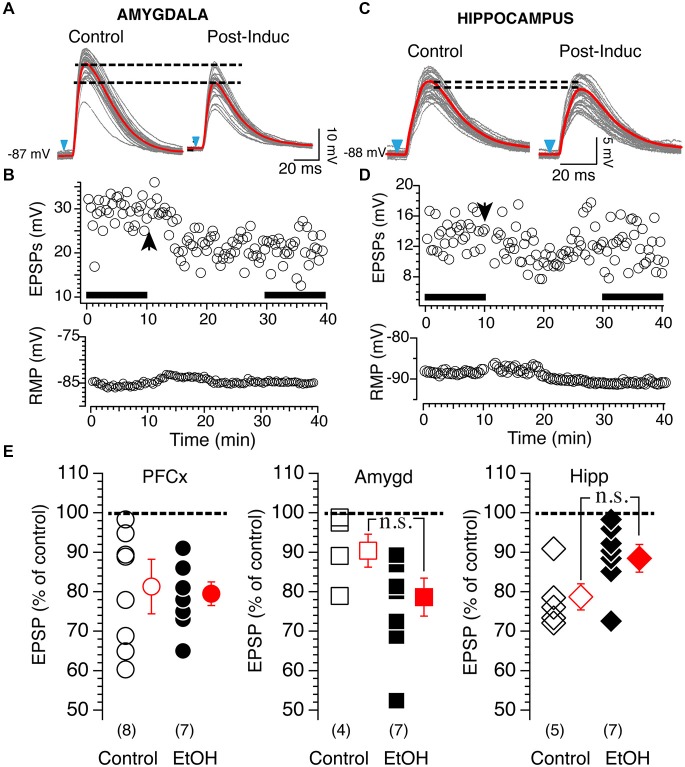
**Effects of 20 mM EtOH on light-evoked plasticity. (A)** Light gray overlapping traces show 30 consecutive EPSPs evoked by stimulating afferents from the amygdala in a representative NAc MSN. EPSPs were recorded at resting membrane potential (−87 mV) before (control) and after (post-induction) AP-EPSP pairing. Broken lines show the difference of averaged EPSP amplitude indicated by red lines, and illustrate a clear depression of the synaptic strength. **(B)** From same neuron as in panel **(A)**, top and bottom graph show EPSPs amplitude and resting membrane potential (bottom graph) monitored every 20 s before and after synaptic plasticity, respectively. Solid bars in top graph indicate when EPSPs shown in panel **(A)** were recorded. **(C)** Light gray overlapping traces show 30 consecutive EPSPs evoked at −86 mV by stimulating afferents from the hippocampus in a representative NAc MSN. EPSPs were recorded before (control) and after (post-induction) AP-EPSP pairing. Broken lines show the difference of averaged EPSP amplitude indicated by red lines, and illustrate a clear depression of the synaptic strength. **(D)** From same neuron as in panel **(A)**, top and bottom graph show EPSPs amplitude and resting membrane potential (bottom graph) monitored every 20 s before and after synaptic plasticity, respectively. Arrowhead indicates time of induction of plasticity. Solid bars in top graph indicate when EPSPs shown in panel **(A)** were recorded. **(E)** Open symbols show amplitude of tLTD evoked in individual MSNs by stimulating cortical (circles, PFCx), amygdala (squares, Amyg) and hippocampal (diamonds, Hippo) pathways. EPSP amplitudes measured 20 min after induction are expressed as percent of control. Solid circles, squares and diamonds indicate EPSPs amplitude recordings following stimulation of cortical, amygdala and hippocampal afferents, respectively, in presence of 20 mM EtOH. Open and solid red symbols show average changes in control and in presence of EtOH, respectively. In parentheses are numbers of neurons recorded in each condition.

Based on these results, we tested 20 mM EtOH on light-driven synaptic plasticity at cortical, amygdala and hippocampal synapses. Stimulation of PFCx evoked tLTD whose amplitude was similar with (82.25 ± 7.8%, *n* = 8; Figure 8E, left panel black circles) or without EtOH (78.98 ± 3.2%, *n* = 7; Figure 8E, left panel, open circles). In contrast, 20 mM EtOH totally blocked tLTP evoked by stimulation of amygdala pathway but enhanced tLTD as shown in Figure 8A. Although the amplitude of tLTD was larger (79 ± 5.1%, *n* = 7) compared to control condition (91.1 ± 4.6%, n=4), this effect was not significant (*F*_(3,7)_ = 2.48, *p* = 0.11). At hippocampal synapses, we observed a small tLTP in only 1 MSN (113% of control, data not shown in graph), while all other recordings showed a slightly weaker tLTD (Figure 8C) with an average of 88.9 ± 3.3% of control (Figure 8E, right panel, *n* = 7) compared to that found in absence of EtOH (79.1 ± 2.9%; Figure 8E). However the difference between the two groups was not statistically significant (*F*_(4,6)_ = 1.37, *p* = 0.054).

### Role of BK Channels and Action Potentials in Mediating EtOH Effects on tLTP and tLTD

**Figure 9:**
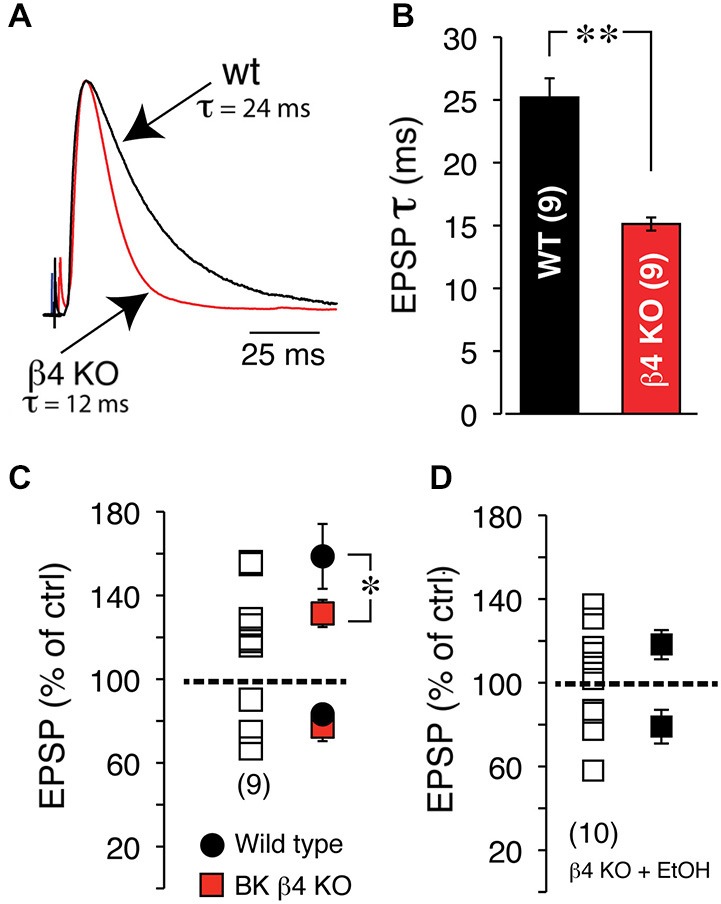
**EtOH inhibits tLTP in part through BK channels. (A)** Representative EPSPs recorded at RMP in wt, (black trace) and BK channel β4 KO mice (red trace). Note the dramatically faster deactivation in KO mice. **(B)** Average deactivation rate in wt (black bar) and KO mice (red bar). **(C)** EPSP amplitude after induction of plasticity in β4 KO mice. Each open square shows amplitude of plasticity in 9 MSNs. Average tLTP (above broken line) and tLTD (below broken line) in wt (solid black circles) and KO mice (solid red squares). **(D)** Effects of acute EtOH on tLTP and tLTD in β4 KO mice.

Since the NMDA receptor is critical for tLTP formation in NAc MSNs (Ji and Martin, 2012), it is very likely the primary target of EtOH. Yet, interaction between this receptor and EtOH cannot fully account for tLTP total inhibition. Indeed, NMDA currents are only partially blocked by EtOH (~50%) in several regions (Weight et al., 1993; Roberto et al., 2006) including the NAc (Nie et al., 1993, 1994) at concentrations much higher than the one used in our optogenetic experiments (i.e., 20 mM). Therefore, tLTP exquisite sensitivity to EtOH points to the involvement of other ion channels. The large conductance calcium- and voltage-gated potassium channel (BK) is a credible candidate for several reasons. Indeed, BKs are abundantly expressed in dendrites of NAc MSNs (Martin et al., 2004), are known to be coupled with NMDA receptors (Isaacson and Murphy, 2001), and their activity is potentiated by EtOH (Dopico et al., 1996), effectively counteracting NMDA-mediated depolarization, and by extension amplifying EtOH’s effects on NMDA receptors. To test this hypothesis, we manipulated the expression of the αβ4 BK channel and examined its role on STDP and EtOH’s effects on tLTP using electrical stimulation to evoke EPSPs. First, we found that BK channels lacking the β4 subunit dramatically accelerated EPSPs deactivation rate from 25.03 ± 1.4 in wt to 15.1 ± 0.45 ms in Knockout (KO) mice (Figures 9A,B), indicating that BK channels shape EPSPs and are likely recruited during EPSP-mediated depolarization. To further probe the role of these channels, we first tested their influence on tLTP and assessed their influence on EtOH-mediated inhibition of tLTP. Thus, compared to wt mice, tLTP in KO mice was significantly reduced (red square, Figure 9C), indicating that BK channels contribute to tLTP formation, probably through their interaction with NMDA receptors. Importantly, we also found that in KO mice, acute EtOH failed to fully inhibiting tLTP (119 ± 4.8% of control, solid square, *n* = 6, Figure 9D), contrary to what we observed in wt mice (Figures 7, 8). These data suggested that, in addition to targeting NMDA receptors, EtOH likely inhibits tLTP in part by enhancing MSNs BK channels activity.

**Figure 10:**
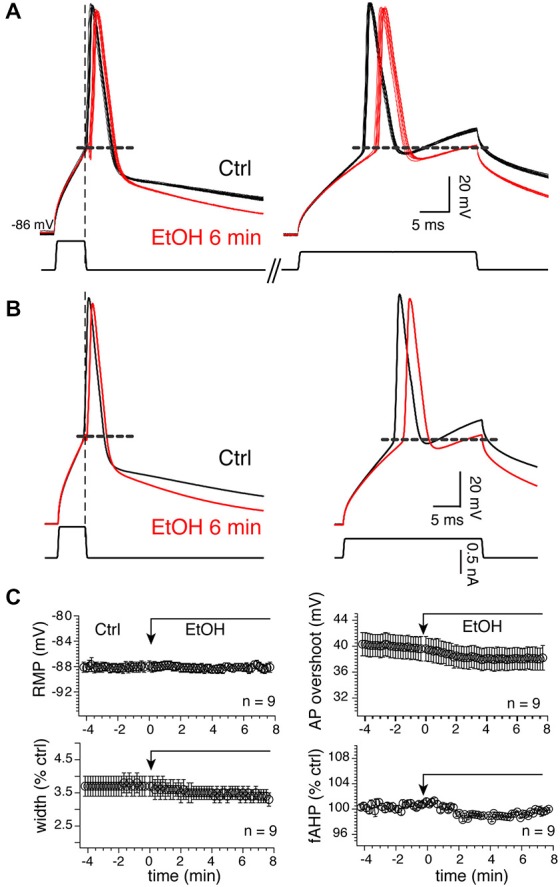
**Twenty millimolar EtOH alters AP properties. (A)** Fifteen consecutive overlapping APs evoked with a short (left) or long (right) injected current pulses before (dark lines) and 6 min (red lines) after EtOH exposure in a representative MSN. Self- (left traces) and current-sustained APs (right traces) were evoked sequentially in the same sweeps. Vertical and horizontal broken lines indicate the end of the short pulse and spike threshold, respectively. **(B)** Average of traces shown in **(A)**. **(C)** Mean ± SEM of resting membrane potential (RMP), AP width (width), amplitude (ampl) and fAHP before (Ctrl) and during EtOH exposure. Except for RMPs, all parameters were expressed as percent of control of values (100%) measured before EtOH perfusion. *p < 0.05 and **p < 0.001.

Because APs control tLTD in MSNs (Ji and Martin, 2012), confirming a previous finding in the dorsal striatum (Shindou et al., 2011), we tested their sensitivity to 20 mM EtOH considering the lack of significant effect of the drug on tLTD. To verify that the effects of alcohol were not altered by injected current, and to ensure that APs were also evoked in conditions similar to those required for induction of plasticity where brief 5 ms current pulses are used, we consecutively evoked APs in two different conditions, first with a short (5 ms) and large current pulse designed to evoke “self-sustained” APs. With the second protocol, we classically evoked APs during depolarizing pulse (“current-sustained”; Figure 10A, right panel). We found that in 9 MSNs, EtOH’s effects on APs were overall very modest as it reduced the duration of “current-sustained” APs by 7.3 ± 1.1% from 3.7–3.4 ms. Similarly, it moderately reduced APs amplitude overshoot by 4.7 ± 1.6% from 39.98–38.01 mV (Figures 10A–C, upper right graph), effects that were not significant. In the remaining four MSNs, we observed the opposite effects as APs duration and amplitude increased by 7.64 and 4.36% in presence of EtOH, respectively. EtOH effects on “self-sustained” APs were similar (data not shown). Taken together, these data indicate that tLTD, in sharp contrast to tLTP, is a form of plasticity that is weakly sensitive to low EtOH concentrations, a result that likely stems from EtOH’s lack of significant effects on APs.

## Discussion

### Origin of Synaptic Inputs Determines Plasticity

In a previous study, we recorded both tLTP and tLTD under the same experimental conditions (i.e., same pairing protocol; Ji and Martin, 2012), a result in sharp contrast to experiments conducted in the cortex, hippocampus and other brain regions, including the dorsal striatum, where changing the direction of plasticity requires reversing AP EPSP pairing order during induction (Feldman, 2012). We initially hypothesized that this result was caused by simultaneous activation of all inputs. To our surprise, selective activation of hippocampal and amygdala inputs still led to both tLTP and tLTD, with their amplitude matching what we previously reported. More intriguing was the fact that AP-EPSP pairing during induction at PFCx synapses totally failed to evoke tLTP, mirroring plasticity at excitatory synapses on GABAergic neurons in a cerebellum like structure in the electric fish (Bell et al., 1997). Similarly, LTP was absent at glutamatergic synapses on GABAergic neurons in the ventral tegmental area, while it was readily evoked at glutamatergic inputs onto dopaminergic neurons in the same brain region, clearly indicating that all glutamatergic synapses are not equal (Bonci and Malenka, 1999). However, a more appropriate comparison is the work of Fino et al. (2005) in MSNs of the dorsal striatum where, despite its strong anatomical similarities with core NAc (Groenewegen et al., 1999), STDP stimulation paradigm evoked tLTP when APs precede EPSPs, and tLTD when the pairing order was reversed. However, despite the claim that they stimulated PFCx afferents, given the non-specific nature of electrical stimulation, they more likely recruited axons irrespective of their origin, making comparison with our data difficult. To further probe plasticity at PFCx inputs, we reversed the pairing order (i.e., EPSP-AP), a configuration that still failed to evoke tLTP, highlighting the notion that cortical synapses cannot undergo LTP on their own, if at all. To explain the absence of tLTP at PFCx synapses it is worth considering that NAc tLTP, unlike tLTD, depends on NMDA receptors (Ji and Martin, 2012), as in almost all cell types studied. Therefore, one possibility is that NMDA receptors at PFCx synapses are different from those at amygdala and hippocampal inputs where tLTP was readily evoked. In particular, a dramatically higher sensitivity to Mg^2+^ may weaken NMDA channel opening and prevent tLTP by lowering intracellular calcium concentrations. However, this idea does not seem supported by our findings showing that Mg^2+^ sensitivity of NMDA receptor at hippocampal synapses, where robust tLTP is observed, is strong. Another possibility may lie upstream with AMPA receptors that are first activated by glutamate, and whose role is to weaken blockade of NMDA receptors by Mg^2+^. Again, we found no difference between AMPA receptors at PFCx and amygdala synapses that could account for the lack of tLTP. In addition to timing, other factors (e.g., membrane depolarization, firing rate) known to contribute to plasticity may be critical. Among these is cooperativity, a term underscoring the need for a minimal number of presynaptic fibers to be simultaneously activated to elicit LTP. Alternatively, contribution of the hippocampal pathways may be necessary for tLTP to happen by providing the needed depolarization that would be rapidly transmitted through the dendritic tree to neighboring PFCx synapses, a priming effect generally known as associativity. Of these two ideas, the latter seems to be anatomically supported by data in rat NAc showing convergence of PFCx and hippocampal inputs on the same dendritic segment (French and Totterdell, 2002), and by *in vivo* intracellular recordings providing evidence that hippocampal inputs gate PFCx information (O’Donnell and Grace, 1995; Mulder et al., 1998). Of note, the presence and absence of tLTP at different synapses is not a phenomenon specific to NAc MSNs as a similar phenomenon was observed in neurons of the lateral nucleus of the amygdala where STDP was present and absent at thalamic and cortical synapses, respectively (Humeau et al., 2005). To explain the lack of tLTP at cortical synapses it is also worth considering the influence of the pairing protocol as a parameter determining synaptic plasticity as shown in cortical pyramidal neurons (Sjöström et al., 2001). While our previous data does not seem to support this idea (Ji and Martin, 2012), we cannot exclude that pairing a short high frequency burst of APs with EPSPs may overcome the tLTP deficit observed in the present study. Finally, the lack of tLTP at cortical synapses is unlikely to reflect a difference of channelrhodopsin-2 (ChR2) expression since EPSPs/Cs amplitude was similar for all inputs. Although the origin of cortical, amygdala and hippocampal inputs to the core and shell subregions are slightly different (Humphries and Prescott, 2010), it is reasonable to wonder whether such differences could affect plasticity in the shell subregion compared to the core.

### Selective Effects of EtOH on Plasticity

In the present study, we report that acute EtOH differentially modulates tLTP in a dose-dependent manner, while affecting tLTD moderately, consistent with studies performed in dorsal striatum (Lovinger et al., 1989; Popp et al., 1998; Yin et al., 2007; Clarke and Adermark, 2010). However, comparison with findings in NAc shows two main differences. First, acute EtOH totally blocks classical NMDA-driven LTD (Jeanes et al., 2011). Regarding LTP, a recent study by Mishra et al. (2012) shows a weak effect only visible at high EtOH concentration (i.e., 50 mM), differences that may stem from very different induction protocols. In our study, the mechanism underlying inhibition of tLTP by EtOH likely primarily involves NMDA receptors that also control tLTP in NAc (Ji and Martin, 2012). As previously mentioned, tLTP high sensitivity to acute EtOH is rather intriguing. In addition to NMDA receptors, our data indicate that at least BK channels contribute to tTLP inhibition by EtOH. However, this result does not mean that other ion channels equally participate to this phenomenon. For example, SK channels, that share some similarities with BKs, and voltage-gated calcium channels represent a viable alternative to BKs as mediating the effects of EtOH (Brodie et al., 2007; Mulholland et al., 2009). Finally, voltage-gated calcium channels may similarly mediate EtOH action on tTLP as these channels are recruited during induction of plasticity and more importantly, are also inhibited by acute EtOH (Wang et al., 1991). Regarding EtOH and tLTD, we found that only hippocampal tLTD was significantly, albeit moderately, inhibited. This is an intriguing finding considering that EtOH altered APs properties in the majority of MSNs tested.

### Functional Implications of Segregated Plasticity

One of the most striking findings of this study is the absence of LTP at cortical synapses, raising the concern that our experimental conditions reflect poorly those found in freely moving animals. Although firing rate of PFCx pyramidal neurons *in vivo* varies, its baseline fluctuates remarkably little around 1 Hz, a frequency comparable to the one used in our study to induce plasticity (Chang et al., 1998; Maurice et al., 1999; Puig et al., 2003). This feature of PFCx pyramidal cells makes it likely that absence of LTP at cortical synapses does reflect physiological conditions. The other intriguing finding is EtOH’s lack of effects on PFCx synaptic plasticity, suggesting that acute effects of EtOH on messages processed by NAc MSNs are not uniform and target primarily contextual and emotional signals coming from the amygdala and hippocampus while leaving cognitive function unaffected. However, as pointed out above, it is possible that tLTP at cortical synapses requires priming by hippocampal and/or possibly amygdala inputs. The functional implications of EtOH selective inhibition of tLTP are unclear at the present time. Yet, efforts at establishing a causal link between LTP/LTD and memory may offer an clue. Although this link remained elusive for a number of years, it has recently been established in mice during fear conditioning (Nabavi et al., 2014). In this study, using optogenetics, LTP and LTD reactivated and inactivated memory formed during fear conditioning, respectively. To the extent that these findings also apply to the accumbens, this would suggest that targeting tLTP, EtOH would prevent the reinstatement of memories or formation of new ones. In light of these findings, it appears that EtOH’s effects on synaptic plasticity are more complex than previously thought, and further investigations are needed to better evaluate the modulation of synaptic plasticity by acute and chronic alcohol in mice NAc.

## Conflict of Interest Statement

The authors declare that the research was conducted in the absence of any commercial or financial relationships that could be construed as a potential conflict of interest.
